# JOINT for large-scale single-cell RNA-sequencing analysis via soft-clustering and parallel computing

**DOI:** 10.1186/s12864-020-07302-6

**Published:** 2021-01-11

**Authors:** Tao Cui, Tingting Wang

**Affiliations:** 1 Department of Pharmacology and Physiology, Georgetown University Medical Center, Washington, DC 20057 USA; 2 Interdisciplinary Program in Neuroscience, Georgetown University Medical Center, Washington, DC 20057 USA

**Keywords:** RNA-Seq, Single-cell, Dropout, JOINT, Deep learning, Probability, Soft-clustering, DEG, Parallel computing

## Abstract

**Background:**

Single-cell RNA-Sequencing (scRNA-Seq) has provided single-cell level insights into complex biological processes. However, the high frequency of gene expression detection failures in scRNA-Seq data make it challenging to achieve reliable identification of cell-types and Differentially Expressed Genes (DEG). Moreover, with the explosive growth of single-cell data using 10x genomics protocol, existing methods will soon reach the computation limit due to scalability issues. The single-cell transcriptomics field desperately need new tools and framework to facilitate large-scale single-cell analysis.

**Results:**

In order to improve the accuracy, robustness, and speed of scRNA-Seq data processing, we propose a generalized zero-inflated negative binomial mixture model, “JOINT,” that can perform probability-based cell-type discovery and DEG analysis simultaneously without the need for imputation. JOINT performs soft-clustering for cell-type identification by computing the probability of individual cells, i.e. each cell can belong to multiple cell types with different probabilities. This is drastically different from existing hard-clustering methods where each cell can only belong to one cell type. The soft-clustering component of the algorithm significantly facilitates the accuracy and robustness of single-cell analysis, especially when the scRNA-Seq datasets are noisy and contain a large number of dropout events. Moreover, JOINT is able to determine the optimal number of cell-types automatically rather than specifying it empirically. The proposed model is an unsupervised learning problem which is solved by using the Expectation and Maximization (EM) algorithm. The EM algorithm is implemented using the TensorFlow deep learning framework, dramatically accelerating the speed for data analysis through parallel GPU computing.

**Conclusions:**

Taken together, the JOINT algorithm is accurate and efficient for large-scale scRNA-Seq data analysis via parallel computing. The Python package that we have developed can be readily applied to aid future advances in parallel computing-based single-cell algorithms and research in various biological and biomedical fields.

**Supplementary Information:**

The online version contains supplementary material available at 10.1186/s12864-020-07302-6.

## Background

scRNA-Seq technology has significantly advanced the understanding of human disease and underlying biological processes at the single-cell level [1, 2]. This ever-evolving technique has revealed cell lineage [3], cell-type heterogeneities [4, 5], and distinct patterns of gene expression [6] that cannot be identified by conventional bulk cell analysis. Despite the rapid growth and maturation of the technique, many experimental and computational challenges remain [7]. Due to the limited amount of RNA extracted from each cell and various technical factors [8], e.g. amplification bias and low RNA capture rate, scRNA-Seq data are very noisy and contain frequent gene expression detection failures (i.e. dropout events [9]). Although several scRNA-Seq imputation methods such as MAGIC [10], scImpute [11], and Saver [12] have been developed to improve analytical accuracy, over-processing of data can cause information loss, and increase the lower bound of detection-error probability due to data processing inequality and Fano’s lemma in information theory [13] (see Methods). Moreover, the massive size of scRNA-Seq datasets demands extensive processing time, hindering the applicability of imputation methods to ever-growing collections of scRNA-Seq data [14]. Together, these challenges significantly hinder the progress of scRNA-Seq in its use as a technique and its application to biological and biomedical research.

Traditional single-cell data processing methods typically perform cell-type identification followed by subsequent DEG analysis [15–17]. However, there are major disadvantages with this two-step method. First, cell-type identification or cell-clustering accuracy may significantly impact DEG analysis. Second, potential valuable information derived from DEG algorithms is not used in cell-type identification. Here, we propose a generalized zero-inflated negative binomial mixture model, “JOINT,” that can perform probability-based cell-type discovery and DEG analysis simultaneously without the need for imputation. The proposed model is an unsupervised learning problem which is solved by using the EM algorithm. Most published studies do not provide test results for model validation, and the statistical distribution of single-cell data remains unclear. We show for the first time (by a statistical test) that the excessive zero-counts in scRNA-Seq data can be explained by this model.

Moreover, JOINT performs soft-clustering for cell-type discovery by computing the probability of cell identity for individual cells, where each cell can belong to multiple cell types with different probabilities. This is different from existing algorithms which typically perform hard-clustering where each cell can only belong to one cell type. JOINT identifies the optimal number of cell-types through Akaike Information Criterion (AIC) automatically rather than specified empirically. All parameters in JOINT are calibrated automatically, without the need for setting hyperparameters, e.g. number of cell-types. Existing clustering algorithms typically perform log-transformation on the count data first, whereas JOINT uses the raw count data directly. Therefore, potential biases introduced during data processing are greatly reduced. We comprehensively evaluated the impact of dropout probability and tested the performance of JOINT on cell-clustering and DEG analysis using simulated and real scRNA-Seq datasets. We show that JOINT obtains better clustering performance on both simulated and real, large-scale scRNA-Seq datasets when compared to existing algorithms.

We also leverage parallel computing methods in data processing: A Python package is implemented and run on GPU using the TensorFlow deep learning framework’s (http://www.tensorflow.org/) low-level API to solve our unsupervised learning model. The computational speed of the JOINT algorithm is 3532 times faster when run on a GPU, versus a Python NumPy implementation on CPU for a simulated dataset with 1000 cells and 2000 genes. We use instructions from TensorFlow directly instead of high-level neural networks APIs such as Keras (https://keras.io/). The Python package that we have developed is the first that can perform cell-clustering and DEG analysis simultaneously on GPU, which dramatically accelerates the computational speed for large-scale scRNA-Seq data analysis. Although not required by JOINT for cell-type identification or DEG analysis, an imputation algorithm is embedded for data visualization.

Finally, our DEG analysis algorithm directly applies soft-clustering results from JOINT, rendering the ability to extract high quality cell-type information and perform accurate DEG identification. Existing GPU-based imputation algorithms only use GPU in the imputation step and still require standard cell-clustering and DEG pipeline in downstream data analysis, which are typically performed on CPU. In contrast, our model does not require the imputation step and can perform both cell-clustering and DEG analysis on GPU. Our study shows a new paradigm of leveraging the use of GPU on large-scale scRNA-Seq data analysis. Overall, the JOINT algorithm provides a more accurate, robust, and scalable method for analysis of large-scale scRNA-Seq datasets. The package that we developed is generic and can be readily applied to aid future advances in parallel computing-based single-cell algorithms.

## Results

### Overview and validation of the JOINT algorithm

**Fig. 1:**
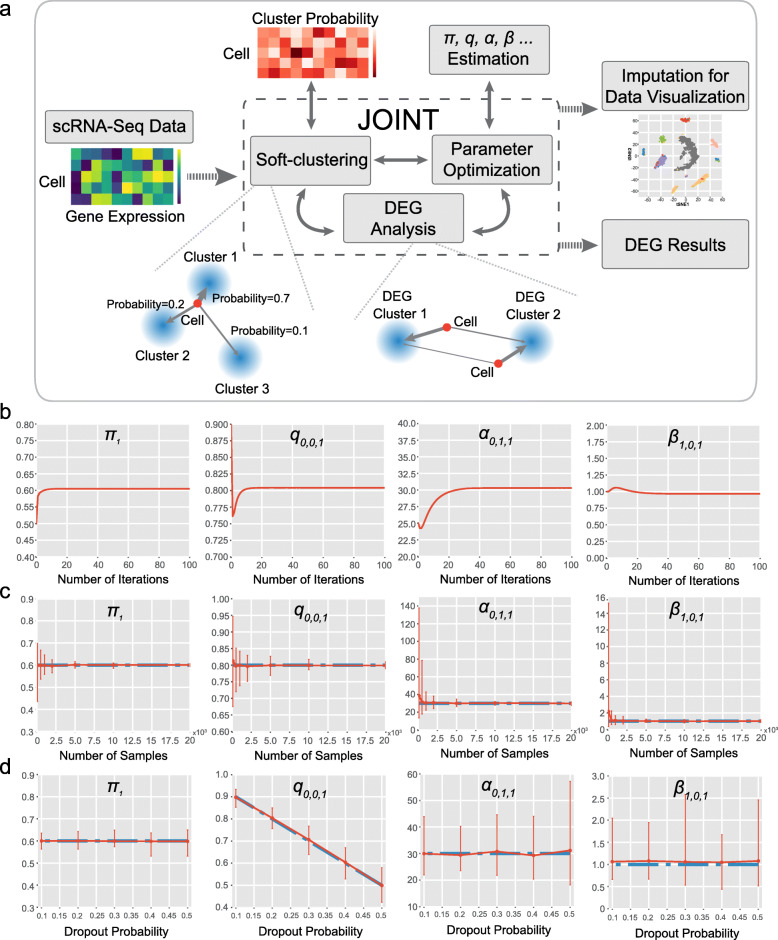
Overview and convergence tests for the JOINT algorithm. **a** Workflow of the JOINT procedure. Soft-clustering, parameter optimization and DEG analysis are performed simultaneously in JOINT. Probability-based soft-clustering for cell-type identification and DEG analysis are demonstrated in the insets. **b** Convergence of *π*_*k*_ (*k* = 1), *q*_*g,k,l*_ (*g* = 0, *k* = 0, and *l* = 1), *α*_*g,k,l*_ (*g* = 0, *k* = 1, and *l* = 1), and *β*_*g,k,l*_ (*g* = 1, *k* = 0, and *l* = 1) to true values with iterations. **c** Convergence of *π*_*1*_, *q*_*0,0,1*_, *α*_*0,1,1*_, and *β*_*1,0,1*_ to true values with the number of samples. **d** Convergence of *π*_*1*_, *q*_*0,0,1*_, *α*_*0,1,1*_, and *β*_*1,0,1*_ to true values with dropout probabilities. True values are indicated by blue lines. Error bars in (**c**) (**d**) indicate the full range of data variation

Existing bulk DEG analysis algorithms (e.g. DESeq2 [18]) and single-cell DEG analysis algorithms (e.g. MAST [19]) assume that cell-type is given, and DEG detection is performed within these given cell-types. As such, cell-type accuracy significantly impacts DEG detection and analysis. Additionally, parameters derived from DEG algorithms may provide useful information for cell-type discovery. We investigate whether simultaneously performing cell-type identification and downstream DEG model calibration benefits both processes. In the JOINT algorithm, we consider the probability of observing count *x* follows a general mixture model. We assume that each mixture component takes a generalized zero-inflated negative binomial model with multiple negative binomial components (see Methods). Instead of performing hard-clustering for cell-type identification, where a given cell is clustered into a particular cell-type, we obtain the probability of individual cells belonging to each cell-type with JOINT. The probability of observing count *x* from cell-type *k* and model parameters are calibrated jointly for cell-type discovery and DEG analysis, rather than fixing cell-type first and estimating DEG parameters thereafter (Methods and Fig. 1a). For each cell-type *k* and gene *g*, our model extends the current use of zero-inflated negative binomial distribution [20] by allowing multiple negative binomial components rather than one. Additionally, we derive an EM algorithm to calibrate all parameters in the zero-inflated negative binomial model for single-cell data automatically, which can also be used for arbitrary numbers of negative binomial components.

We first validated the model by testing whether it could explain the excessive zero-counts in a real scRNA-Seq dataset. We chose the Zeisel dataset [21] and analyzed gene expression with the “Oligodendrocyte” label provided in the dataset (see Methods). For each gene, we tested the performance of three JOINT variations: 1) *negative binomial* (Poisson-Gamma mixture), 2) *zero-inflated negative binomial*, and 3) *zero-inflated negative binomial with two components*. We trained all three variations of the algorithm on GPU using TensorFlow, obtained predicted zero-count probability for each gene across all cells and compare the mean to the empirical zero-count probability. Then, we tested if the predicted zero-count probability is significantly different than the empirical value for each JOINT variation (see Methods). We found that *p*-values for the comparisons were: *p* = 1.58e^− 19^ for 1) *negative binomial*, *p* = 0.057 for 2) *zero-inflated negative binomial*, and *p* = 1.12e^− 10^ for 3) *zero-inflated negative binomial with two components*. Since the zero-count probability from 2) *zero-inflated negative binomial model* is not significantly different than the empirical value, we concluded that this variation can recover the zero-count probability. This finding provides the first statistical evidence that excessive zero-counts in scRNA-Seq data can be explained by a zero-inflated negative binomial distribution. In the rest of the paper, we assume that gene expression follows the zero-inflated negative binomial distribution (with one component), but arbitrary numbers of negative binomial components can be selected and applied in the model for different single-cell datasets.

Next, as a sanity test, we examined whether the JOINT algorithm can converge to true values. We generated a simulated dataset with two cell-types (clusters) and two genes as the “ground truth” (see Methods). JOINT successfully converged to true values when we varied the number of iterations, number of samples (cells), and dropout probabilities (Fig. 1b-d and Fig. S1, S2, S3).

### Evaluation of clustering performance using simulated datasets

**Fig. 2:**
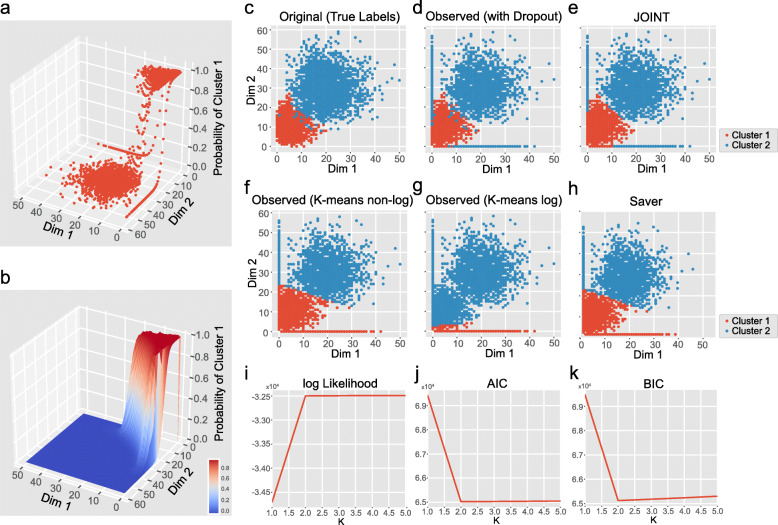
Validation of JOINT’s clustering performance. **a** Cell-clustering by JOINT on a simulated dataset with two cell-types and two genes. Scatter plot shows posterior probability (z-axis) for each cell (red dots) belonging to cell-type 1. Expression levels of gene 1 (Dimension 1, Dim 1) and gene 2 (Dimension 2, Dim 2) are shown on the x- and y-axis. **b** Surface plot shows the probability for individual cells belonging to cell-type 1 (hot color) and 2 (cold color). **c** - **h** Comparison of the clustering performance of different algorithms. **c** Original dataset without dropout (True Labels). **d** Observed dataset with 0.2 dropout probability. **e** Cell-clustering by JOINT on the dataset with 0.2 dropout probability. **f** Cell-clustering by K-means on non-log data with 0.2 dropout probability. **g** Cell-clustering by K-means on log-transformed data with 0.2 dropout probability. **h** Cell-clustering by K-mean on Saver-imputed data (non-log) with 0.2 dropout probability. Individual cells in clusters 1 and 2 are shown in red and blue, respectively. **i** - **k** The JOINT algorithm determines cell-cluster numbers automatically by likelihood (**i**), AIC (**j**), and BIC (**k**) tests

We next compared the clustering performance of JOINT to other algorithms using a simulated dataset containing two cell-types and two genes (Fig. 2 and Table S1). We fixed the dropout probability at *q*_*0*_ = 0.2 and generated 5000 cells (see Methods). For published algorithms, we applied K-means clustering with 100 random initial points to the dataset and chose clustering results with the best Adjusted Rand Score for comparison. We compared the performance of JOINT on the original non-imputed data, to K-means on the non-imputed and Saver [12] -imputed datasets (Fig. 2a-h and Table S1). ScImpute [11] was not included since it cannot be applied to 2-dimensional data. We demonstrated that JOINT obtained much higher clustering scores on the non-imputed data, than K-means on both the non-imputed and Saver-imputed datasets. JOINT’s performance also surpassed that of K-means on the original data without dropout (Table S1). In this dataset, K-means performance was worse in log-transformed counts when compared to non-log-transformed data, suggesting log-transformation may lead to information loss (Fig. 2f and g). In contrast, non-log-transformed raw data can be directly used in the JOINT algorithm, minimizing potential bias and information loss. The JOINT algorithm can also automatically optimize the number of clusters through AIC, rather than forcing a choice from intuition. We ran the JOINT algorithm with the number of clusters *K* ranging from 1 to 5. For each *K*, we randomly chose initial points, ran the proposed JOINT algorithm 10 times, and chose results with the highest likelihood. We found that the log likelihood did not increase when *K* was greater than 2, and both AIC and Bayesian Information Criterion (BIC) were minimized when *K* = 2. Therefore, JOINT took *K* = 2 as the optimal number of clusters, which precisely predicted the number of clusters in the simulated dataset (Fig. 2i-k).

**Fig. 3:**
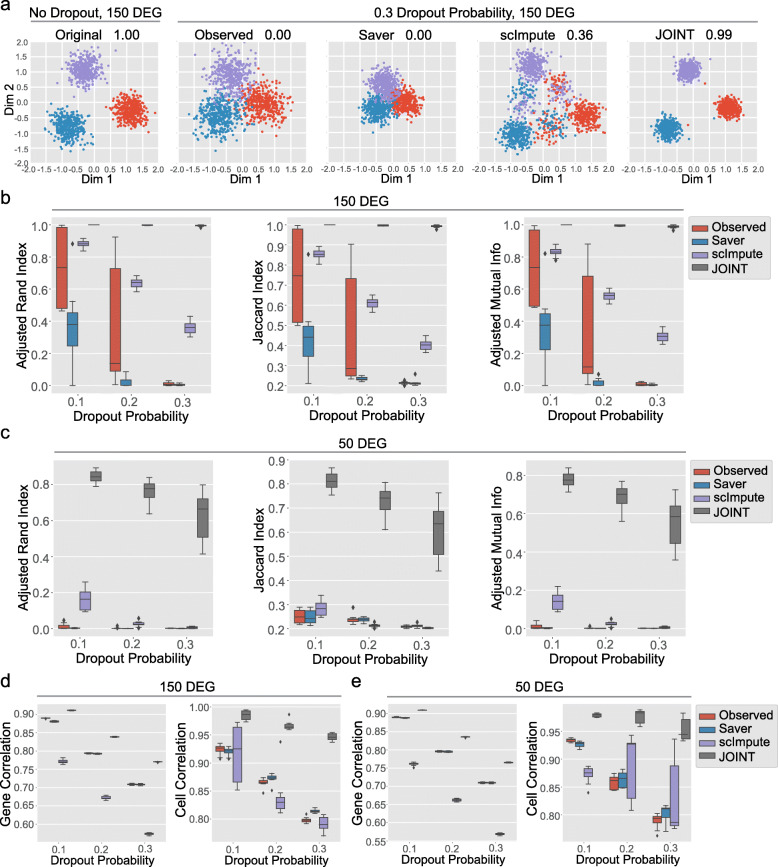
Comparison of clustering performance of different algorithms at various dropout probabilities and DEG numbers. **a** Cell-clustering by JOINT, Saver, and scImpute on a simulated dataset with three clusters (dropout probability is set to 0.3 and DEG number set to 150). Original data with no dropout is shown on the left. Adjusted Rand Index for each algorithm is shown. K-means clustering method is used for published imputation algorithms. Imputation algorithm in JOINT is used for data visualization. For datasets with dropout, we applied the PCA from the original dataset without dropout to get the 2-dimensional plot. **b** - **c** Cell-clustering scores are compared for JOINT, Saver, and scImpute algorithms at different dropout probabilities on a dataset with 150 DEG (**b**) and 50 DEG (**c**). **d** - **e** Correlation coefficients of cell-clustering results from JOINT, Saver, and scImpute to original “true labels” are averaged across all genes (Gene Correlation) or cells (Cell Correlation) at different dropout probabilities. Correlation coefficients generated from a dataset with 150 DEG (**d**) and 50 DEG (**e**) are shown

We further examined JOINT’s performance on a more complex simulated dataset with three cell-types, using parameters derived from published scRNA-Seq data to mimic real experimental settings (Methods and Fig. S4). We systematically examined the clustering performance of JOINT at different dropout probabilities and DEG numbers. We evaluated the performance of JOINT and other published algorithms at dropout probability *q*_*0*_ = 0.1, 0.2 and 0.3 and DEG number *n* = 50, 100 and 150 (Fig. 3 and Fig. S5, S6, S7). We generated 10 datasets for each dropout probability and DEG number combination, and applied JOINT, Saver, and scImpute to each dataset. We showed that JOINT obtained the highest Adjusted Rand Index score among all algorithms tested, strongly suggesting its performance was superior over Saver and scImpute (Fig. 3a-c and Fig. S6a-d). It is worth noting that although JOINT performs cell-type identification without the need of imputation, it acquires the ability to impute for data visualization (Methods, Fig. 3, and Fig. S5, S6, S7).

Finally, we compared the clustering outputs from JOINT, Saver, and scImpute to the original dataset without dropout, to access the accuracy of performance. Since we used a simulated dataset, “true labels” without dropout were known. We correlated the clustering outputs to “true labels,” and compared the correlation coefficients for the different algorithms. Higher correlation coefficients indicate better performance. We found that when we performed this correlation test at different dropout probabilities and DEG numbers, JOINT obtained higher correlation coefficients than other imputation methods (Fig. 3d, e, and Fig. S6e). Overall, we leveraged a simulated dataset with known cell-types to evaluate the performance of JOINT at different dropout probabilities and DEG numbers. Since the simulated dataset was generated using parameters derived from real scRNA-Seq data, we validated the JOINT algorithm in conditions that mimic real experimental settings.

### Evaluation of clustering performance using real, large-scale scRNA-Seq datasets

**Fig. 4:**
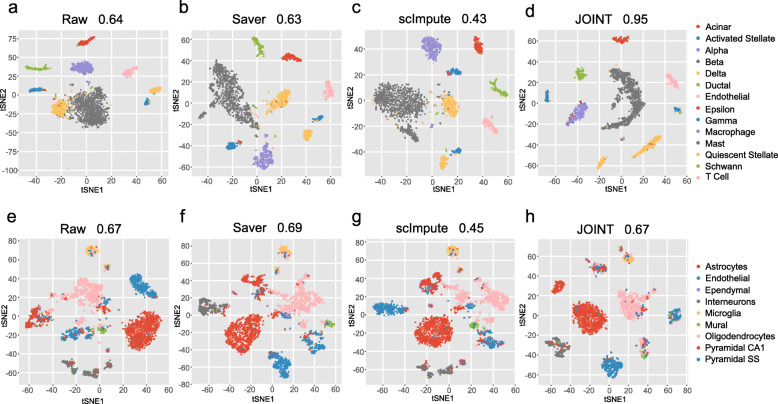
Evaluation of JOINT’s clustering performance with real, large-scale scRNA-Seq datasets. **a** - **d** Cell-clustering and t-SNE visualization of the Barron dataset. Cell-clustering from raw data (**a**), Saver-imputed data (**b**), scImpute-imputed data (**c**), and JOINT (**d**) are shown. Imputation algorithm in JOINT is used to visualize cell-clustering results. Adjusted Rand Index scores are shown for all algorithms. **e** - **h** Cell-clustering and t-SNE visualization of the Zeisel dataset. Cell-clustering from the raw data (**e**), Saver-imputed data (**f**), scImpute-imputed data (**g**), and JOINT (**h**) are shown. Imputation algorithm in JOINT is used to visualize cell-clustering results. Adjusted Rand Index scores are shown for all algorithms

To futher evaluate JOINT’s performance, we compared its clustering performance and computing time to Saver and scImpute using real, large-scale scRNA-Seq datasets (Baron [22] and Zeisel [21]). The cell-types identified by JOINT algorithm matched the published results when applied to the Baron and Zeisel data (Fig. 4d and h). JOINT also obtained higher or comparable Adjusted Rand Index, Jaccard Index, and Adjusted Mutual Information scores when compared to Saver and scImpute methods (Fig. 4 and Table 1).

**Table 1:** Comparison of clustering performance and computing time for JOINT and published imputation algorithms on real scRNA-Seq datasets

Performance Scores	Raw	Saver	scImpute	JOINT
Baron Dataset				
Adjusted Rand Index	0.64	0.63	0.43	0.95
Jaccard Index	0.55	0.53	0.34	0.92
Adjusted Mutual Info	0.79	0.76	0.64	0.89
Zeisel Dataset				
Adjusted Rand Index	0.67	0.69	0.45	0.67
Jaccard Index	0.57	0.59	0.35	0.57
Adjusted Mutual Info	0.63	0.63	0.56	0.65
Computing Time (s)	Saver	scImpute	JOINT	
Baron Dataset	4777	1010	528	
Zeisel Dataset	18036	3440	836	

We then evaluated the computing time of JOINT compared to other imputation algorithms. We found both performance and speed of the JOINT algorithm were dramatically accelerated over existing algorithms (Table 1). This is the first study that systematically examined the performance and computing time of different imputation algorithms. The JOINT algorithm functions as a useful parallel computing-based method for scalable scRNA-Seq analysis. Since JOINT runs from an initial point, we also examined whether clustering performance was improved by the EM algorithm through JOINT, or relied heavily on initial conditions. We compared the JOINT-obtained clustering scores on the Zeisel dataset using randomly selected initial points or those selected through K-means with and without the application of EM algorithm. We demonstrated that the EM algorithm indeed improved the clustering performance of JOINT when the initial points were either randomly selected or using K-means (Fig. S8).

### Evaluation of JOINT performance in DEG analysis

**Fig. 5:**
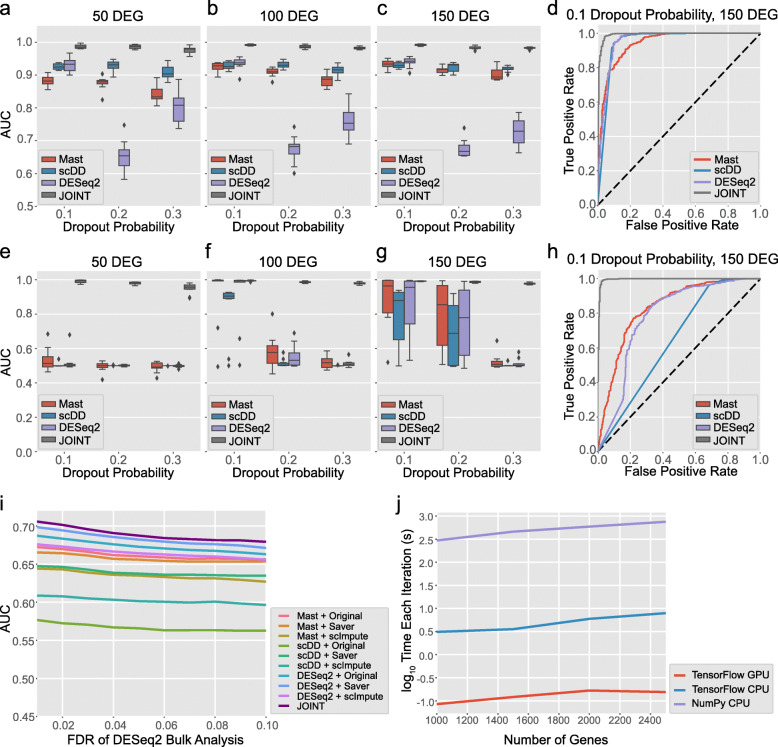
Evaluation of JOINT’s performance in DEG analysis. **a** - **d** Comparison of the performance of DEG analysis algorithms when cell labels are known and different dropout probabilities are assigned to each cell-cluster. AUC scores for MAST, scDD, DESeq2, and JOINT when different dropout probabilities are assigned to each cell-cluster in datasets with 50 DEG (**a**), 100 DEG (**b**) and 150 DEG (**c**) are shown. **d** ROC curves for MAST, scDD, DESeq2, and JOINT when mean dropout probability for all cells is set to 0.1 (dropout probability varies by 0.05 for each cell-cluster) and DEG number is set to 150. **e** - **h** Comparison of the performance of different DEG analysis algorithms when cell labels are unknown and the same dropout probability is assigned to all cells. AUC scores for MAST, scDD, DESeq2, and JOINT when the dropout probability is set to the same value for all cells in datasets with 50 DEG (**e**), 100 DEG (**f**) and 150 DEG (**g**) are shown. **h** ROC curves for MAST, scDD, DESeq2, and JOINT when mean dropout probability for all cells is set to 0.1 and DEG number is set to 150. **i** AUC curves of DEG analysis algorithms in combination with imputation methods and JOINT are shown. **j** Computing time of one iteration of the JOINT EM algorithm when run by TensorFlow using GPU, TensorFlow using CPU (run on compiled C code), and Python-based NumPy implementation using CPU. Computing time is tested for different numbers of genes

The JOINT algorithm also acquires the function of performing DEG analysis simultaneously with cell-type identification. We evaluated JOINT’s performance in DEG analysis using a simulated dataset with 3 clusters from cells labeled “CA1 Pyramidal” from the Zeisel dataset [21] (see Methods). We examined JOINT’s performance in two conditions: true cell-type labels as known or unknown. First, we assumed that all cell-types were known, and set the dropout probability to *q*_*0*_ = 0.1, 0.2, and 0.3 for all cells and selected *n* = 50, 100, and 150 DEG in the simulated dataset. In real experimental settings, dropout probability is unlikely to be a set number across all cells. Therefore, we varied the dropout probability *q*_*0*_ by 0.05 for each cluster (e.g. When *q*_*0,mean*_ for all cells = 0.1, we obtained *q*_*0*_ = 0.05, 0.1, and 0.15 for clusters 1, 2, and 3 respectively). The performance of JOINT and other published DEG analysis algorithms were evaluated using the false/true positive rate relationship (Receiver Operating Characteristic (ROC) curve). DEG analysis results from cluster 1 and cluster 3 were then compared across algorithms (Fig. 5a-d). When we used Area Under the Curve (AUC [23]) to compare the performance of MAST [19], scDD [24], DESeq2 [18], and JOINT, we found that JOINT obtained higher AUC scores compared to other algorithms at different dropout probabilities and DEG numbers (Fig. 5a-d).

Next, we considered the case where cell-type labels were unknown, but derived from a clustering algorithm. Since cell-types are unknown before analysis in real scRNA-Seq datasets, this test allows us to evaluate all algorithms in conditions similar to real experiments. For published DEG analysis algorithms, we first performed K-means clustering and spectral clustering on log(1 + count), PCA on log(1 + count) with 2 components, and PCA on log(1 + count) with components explaining 25% or 40% of variance on the simulated data. Cluster labels which generate the highest Adjusted Rand Index scores were chosen for DEG analysis for published methods. For JOINT, we initialized the algorithm with the same 8 conditions for fair comparison. We want to emphasize that for existing DEG analysis methods, true cell labels must be known in order to compute Adjusted Rand Index scores. Since we opted to use the highest Adjusted Rand Index scores for published algorithms, it is in fact, an overestimation of their performance. In contrast for JOINT, we chose the clustering results that provided the highest likelihood for individual cells belonging to certain clusters, thus eliminating the need of knowing true cell labels beforehand. Based on the clustering results from each algorithm, we identified cell-types with the highest correlation with the original clusters 1 and 3, and performed DEG analysis on these clusters. We compared AUC scores for MAST, scDD, DESeq2 and JOINT algorithms. We found the JOINT algorithm obtained the best AUC scores among all the DEG analysis methods tested at different dropout probabilities (same dropout probability across all cells) and DEG numbers (Fig. 5e-h).

Finally, we evaluated JOINT’s performance in DEG analysis using a real, large-scale scRNA dataset. We analyzed a scRNA-Seq dataset GSE75748 [25] with both bulk and single-cell RNA-seq data on human embryonic stem cells (ESC) and definitive endoderm cells (DEC). This dataset includes four samples in H1 ESC, and two samples in DEC from bulk RNA-Seq; 212 cells in H1 ESC and 138 cells in DEC from scRNA-Seq. We used an R package (DESeq2) to identify DEG from bulk data and applied MAST, scDD, and DESeq2 to identify DEGs from the original scRNA-seq data or imputed data by Saver and scImpute. As DESeq2 requires non-zero integer inputs, we rounded the imputed counts and added 1 for DEG analysis. We applied different thresholds to False Discovery Rates (FDRs) of genes in bulk data to obtain a DEG list as the reference for single-cell DEG analysis. Next, we compared AUC scores for JOINT and other DEG analysis algorithms in combination with imputation methods. All algorithms that were used for comparison include: MAST+Original, MAST+Saver, MAST+scImpute, scDD+original, scDD+Saver, scDD+scImpute, DESeq2 + Original, DESeq2 + Saver, DESeq2 + scImpute, and JOINT. We found JOINT had superior performance over all other existing imputation and DEG analysis algorithms that were tested (Fig. 5i).

We also systematically examined the computational time of JOINT. We compared the computational time of one iteration in the EM algorithm between TensorFlow using GPU, TensorFlow using CPU (run on compiled C code), and Python-based NumPy implementation using CPU. We examined the scenario with 1000 cells and 9 cell-types. We simulated the dataset randomly and varied the number of genes from 1000 to 2500 (Fig. 5j). When the number of genes is 2000 (based on the number of highly differential genes used in Seurat procedure), we found that TensorFlow run on GPU had a 35.6x speedup over TensorFlow run on CPU, and a 3532x speedup over NumPy run on CPU (Fig. 5j and Table S2). Overall, we demonstrated that the performance of JOINT significantly improved both the accuracy and efficiency of DEG analysis compared to current algorithms.

## Discussion

We propose a mathematical algorithm, “JOINT,” that performs cell-type discovery and DEG analysis by parallel computing. Since there is no need for imputation, the potential for information loss from data over-processing is minimized. Instead of assigning each cell into a hard-cluster, this cell-type probability-based soft-clustering approach makes this algorithm more accurate and robust. We validated the model extensively, and examined the performance of JOINT on cell-type identification and DEG analysis using both simulated and real, large-scale scRNA-Seq datasets. Most published studies do not provide test results for model validation, and the statistical distribution of single-cell data from these models is unclear. We show, for the first time, that excessive zero-counts in real scRNA-Seq data can be explained by a properly trained zero-inflated negative binomial distribution. All parameters in JOINT are calibrated automatically without needing to set any hyperparameters, such as the number of cell-types. While existing clustering algorithms typically perform log-transformation on the count data first, our model uses the raw count data directly. Therefore, potential biases introduced during data processing are greatly reduced. Moreover, when we evaluate the performance of JOINT on cell-type identification and DEG analysis, the joint-analysis feature of JOINT makes it more reliable and efficient over existing algorithms that were tested.

We developed a Python package using the TensorFlow low-level API to train our model on GPU. The computational speed of the JOINT algorithm is 3532 times faster when run on a GPU versus a Python NumPy implementation on CPU for a simulated dataset. The Python package we have developed is the first one that can perform cell-clustering and DEG analysis simultaneously on GPU, which dramatically facilitates an increase in computing speed for large-scale scRNA-Seq data analysis. The Python package is generic and can be applied to a generalized zero-inflated negative binomial distribution with arbitrary number of negative binomial components for different scRNA-Seq datasets.

In conclusion, JOINT can be readily applied to aid future advances in parallel computing-based single-cell algorithms. JOINT greatly improves the accuracy, scalability and speed of single-cell data processing, making it a suitable candidate for future work involving scalable scRNA-Seq data analysis.

## Methods

### Over-processing of data by imputation may cause information loss due to data processing inequality and Fano’s lemma

Let three random variables form the Markov chain *X* → *X*^′^ → *Y*, implying that the conditional distribution of *Y* depends only on *X*^′^ and is conditionally independent of *X*. By data processing inequality [13], the mutual information between *X* and *Y* is greater than or equal to that between *X*^′^ and *Y*, i.e.

1 $$ I\left(X;Y\right)\ge I\left({X}^{\prime };Y\right). $$

*X* is observed single-cell data, *X*^′^ is imputed data, *Y* is decision variables, such as cell-types or DEG. This equation indicates the information of data cannot be increased from data imputation. Note that if we have a priori information *S* about genes or cell-types, we may have $I\left(X;Y\right)\le I\left({X}^{\prime };Y|S\right)$, which indicates data imputation with a priori information may improve mutual information. But even in this case, we still have *I*(*X*; *Y*| *S*) ≥ *I*(*X*′; *Y*| *S*).

From Fano’s inequality, we have a lower bound on the detection-error probability (cell-type mis-classification or DEG mis-detection)

2 $$ {p}_e=\Pr \left(\hat{Y}\ne Y\right)\ge \frac{H(Y)-I\left(X;Y\right)-1}{\log \left(|Y|\right)}. $$

From data processing inequality, if processed data *X*^′^ instead of un-processed data *X* is used, the right-hand side of eq. (2) becomes bigger. Even though (2) is only a lower bound, data imputation increases the lower bound of error-detection. Therefore, performing data imputation on observed data and performing subsequent analysis leads to information loss and an increase of a lower bound on the detection-error probability. This indicates that there is an opportunity to perform cell-type discovery and DEG analysis simultaneously to prevent such an information loss.

### JOINT algorithm

In the JOINT algorithm we consider a general mixture model

$$ p(x)=\sum \limits_{k=0}^{K-1}{\pi}_k{f}_{k\left(\left.x\right|{\theta}_k\right),} $$

where *x* is observed count number, *k* is the number of cell-types, *π*_*k*_ is the probability of choosing cell-type *k* and *f*_*k*_(*x*|*θ*_*k*_) is the probability of observing *x* given parameters *θ*_*k*_ in cell-type *k*. Given *x* and *θ*_*k*_, we compute the posterior probability of observed counts *x* from cell-type *k* as

$$ p\left(\left.k\right|x\right)=\frac{\pi_k{f}_k\left(\left.x\right|{\theta}_k\right)}{\sum_{k=0}^{K-1}{\pi}_k{f}_k\left(\left.x\right|{\theta}_k\right)}. $$

Rather than using hard-clustering methods where a given cell is clustered into a particular cell-type, we obtain the probability of individual cell belonging to each cell-type (Fig. 1a). If a cell has non-zero probability *p* belonging to cell-type *k*, then it contributes accordingly (proportional to *p*) to clustering and DEG analysis for cell-type *k* (Fig. 1a). Here, we assume that *f*_*k*_(*x*|*θ*_*k*_) takes a generalized zero-inflated negative binomial model with multiple negative binomial components

$$ {q}_{g,k,0}{1}_{x_g==0}+\sum \limits_{l=1}^{L-1}{q}_{g,k,l}\int Gamma\left({\lambda}_{g,k,l}|{\alpha}_{g,k,l},{\beta}_{g,k,l}\right) Poisson\left({x}_g|{s}_c{\lambda}_{g,k,l}\right)d{\lambda}_{g,k,l}, $$

where there are *L* components, *q*_*g*, *k*, 0_ is the dropout probability for gene *g* in cell-type *k*, ${1}_{x_g==0}$ is 1 when *x*_*g*_ = 0, and otherwise 0. *q*_*g*,*k*,*l*_ is the probability that the observed count *x*_*g*_ is from the *l*-th negative binomial component for gene *g* in cell-type *k*, and *s*_*c*_ is a cell level scaler. We choose the same cell scaler as Seurat process which normalizes the library size to 10,000. The dropout probability *q*_*g*,*k*,0_ is the probability of observing zero-counts, regardless of the real expression level of gene *g*. When the first dropout term is omitted and *L* = 1, we obtain a *negative binomial model*. When *L* = 2, the model reduces to the *zero-inflated negative binomial model*. When *L* = 3, we obtain a *zero-inflated negative binomial model with two components*. Note that *f*_*k*_(*x*|*θ*_*k*_) can be also adapted and used for other models in DEG analysis.

To generate observed count *x*, we first draw a cell-type *k* from *π*, which determines a set of parameters used for each gene in cell-type *k*. Then, we choose a negative binomial component type *l* with probability *q*_*g,k,l*_. When *l* = 0, we set *x*_*g*_ = 0, which corresponds to dropout and the process stops. When *l* > 0, we choose *α*_*g,k.l*_ and *β*_*g,k.l*_ for each gene in cell-type *k* and generate a Poisson intensity λ_*g,k,l*_. Finally, we generate the observed count *x*_*g*_ from a Poisson distribution with intensity λ_*g,k,l*_. Given observed counts in a given cell *x* = [*x*_0_, …, *x*_*G − 1*_], we estimate *θ* = {*α*_*g*,*k*,*l*_, *β*_*g*,*k*,*l*_, *q*_*g*,*k*,*l*_, *π*_*k*_} by maximizing the Probability Mass Function

$$ p\left(x|{\pi}_k,{q}_{g,k,l},{\alpha}_{g,k,l},{\beta}_{g,k,l}\right)=\sum \limits_{k=0}^{K-1}{\pi}_k\prod \limits_{g=0}^{G-1}\left({q}_{g,k,0}{1}_{x_g==0}+\sum \limits_{l=1}^{L-1}{q}_{g,k,l}\int Gamma\left({\lambda}_{g,k,l}|{\alpha}_{g,k,l},{\beta}_{g,k,l}\right) Poisson\left({x}_g|{s}_c{\lambda}_{g,k,l}\right)d{\lambda}_{g,k,l}\right), $$

where we assume individual genes obtain independent parameters *α*_*g*,*k*,*l*_, *β*_*g*,*k*,*l*_, *q*_*g*,*k*,*l*_.

We do not assume a constant dispersion across all genes but rather each gene has its own *α*_*g*,*k*,*l*_ and *β*_*g*,*k*,*l*_. The dropout probability *q*_*g,k,0*_ is optimized for each gene without assuming specific dependence on the mean expression. Each cell-type has its own negative binomial distribution rather than a single distribution shared across all cell-types. The mixture model is an unsupervised learning problem which is solved using the EM algorithm.

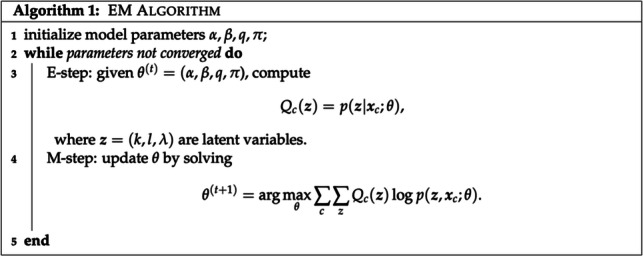



The probability of *x* from cell-type *k* and negative binomial distribution parameters *α*_*g*,*k*,*l*_ and *β*_*g*,*k*,*l*_ (also used for DEG analysis) are calibrated jointly, rather than fixing the cell-type first and estimating parameters for DEG analysis thereafter. Although usually challenging when run on CPU especially with big dataset, model calibration is successfully achieved when it is trained on GPU. All model training and testing was performed on a computer with Intel Xeon CPU E5–2686 v4 @ 2.30GHz with 62GB RAM and NVIDIA Tesla K80 GPU with 17GB memory.

### Model validation using the Zeisel dataset

We chose the Zeisel dataset [21] and analyzed the gene expression with the “Oligodendrocyte” label provided in the dataset for model validation. Top and bottom 10% cells were removed based on their library size. Genes that have non-zero expression between 30 and 90% were chosen. This resulted in a dataset with 742 cells and 3069 genes for model testing and validation. For each gene, we tested the performance of three variations of the JOINT algorithm: 1) *negative binomial* (Poisson-Gamma mixture), 2) *zero-inflated negative binomial* (initial points were: dropout probability *q*_*0*_ = 0.1, *α* = mean, and *β* = 1), 3) *zero-inflated negative binomial with two components* where one component started from *α* = 0.1 and *β* = 1 (mimic a Poisson component with rate 0.1 from reference [23]) and the other one started from *α* = mean and *β* = 1 in training. The initial probability *q*_*0*_ was set to 0.5 for the first and 0.4 for the second components. For the proposed generalized zero-inflated negative binomial model with multiple negative binomial components, the probability of getting zero-count is

$$ {q}_{g,k,0}+\sum \limits_{l=1}^{L-1}{q}_{g,k,l}{\left(\frac{\beta_{g,k,l}}{\beta_{g,k,l}+{s}_c}\right)}^{\alpha_{g,k,l}}. $$

In order to test whether the three variations of JOINT algorithm can explain the zero-counts in the Zeisel dataset, we trained all three variations of the algorithm on GPU using TensorFlow, obtained predicted zero-count probability ${\hat{p}}_{c,g}^0$ for each gene *g* and cell *c*, then calculated the mean across all cells for each gene ${\hat{p}}_g^0=\frac{1}{C}\sum {\hat{p}}_{c,g}^0$. We compared ${\hat{p}}_g^0$ to the empirical zero-count probability for each gene ${\overline{p}}_g^0$ by counting the number of cells with zero-count (for this gene), divided by the total number of cells. Then, we performed two-sided student t-tests with the null hypothesis that ${\hat{p}}_g^0-{\overline{p}}_g^0$ has mean 0, to examine whether each variation of the model can recover the zero-count probability. We found that *p*-values were: *p* = 1.58e^− 19^ for *negative binomial*, *p* = 0.057 for *zero-inflated negative binomial*, and *p* = 1.12e^− 10^ for *zero-inflated negative binomial with two components*. Since we could not reject the null hypothesis (i.e. predicted zero-count probability is the same as the empirical estimate at 95% confidence level), we concluded that the *zero-inflated negative binomial model* can recover the zero-count probability. Although model 3 subsumes model 2, the EM algorithm may converge to a suboptimal local optimum when model 3 is initialized as in Methods.

### Generation of a simulated dataset with two genes and two cell-types

#### Simulation set up

In order to validate and test the clustering performance of the model (Fig. 1b-d, Fig. 2, Fig. S1, S2, S3 and Table S1), we generated a simulated dataset with two genes and two cell-types (clusters) as the “ground truth.” To set up the simulation, we chose *π* = {0.4,0.6}, *q*_*g,k,0*_ = 0.2, *q*_*g,k,1*_ = 0.8, and *β*_*g,k,1*_ = 1.0; first cluster *α*_*0,0,1*_ = 10 and *α*_*1,0,1*_ = 5; second cluster *α*_*0,1,1*_ = 30 and *α*_*1,1,1*_ = 20.

#### Convergence of the model with iterations

We generated 10,000 samples from the mixture model using parameters described above. In the EM algorithm, we chose initial values *π* = {0.5,0.5}, *q*_*g,k,0*_ = 0.1, *q*_*g,k,l*_ = 0.9, and *β*_*g,k,l*_ = 1.0; first cluster *α*_*0,0,1*_ = 8 and *α*_*1,0,1*_ = 8; second cluster *α*_*0,1,1*_ = 25 and *α*_*1,1,1*_ = 25. The JOINT algorithm converged after 30 iterations (Fig. 1b and Fig. S1).

#### Convergence of the model with number of samples

For a given number of samples, we randomly generated 50 datasets and applied JOINT on each dataset for statistics. As the number of samples increased, we found that the EM estimate converged to the actual values with smaller variances (Fig. 1c and Fig. S2). This agrees with the fact that Maximum Likelihood (ML) estimates converge almost surely to true values asymptotically when the number of samples goes to infinity [26].

#### Convergence of the model with dropout probability

We fixed the number of samples as 1000 and varied the dropout probability *q*_*g,k,0*_ from 0.1 to 0.5 with step size of 0.1. At each dropout probability, we generated 50 datasets and ran JOINT on each dataset to test the convergence (Fig. 1d and Fig. S3).

### Generation of a simulated dataset with three cell-types using Zeisel data

We simulated a scRNA-Seq dataset with 3 cell-types (Fig. 3 and Fig. S5, S6, S7). We trained JOINT on cells with the “CA1 Pyramidal” label in the Zeisel dataset [21] for each gene using the EM algorithm. First, we chose cells with > 10,000 library size and genes with non-zero-counts in at least 40% of cells. Then, we trained the JOINT algorithm on the 3529 genes and 834 cells that were selected. Next, we randomly chose 1000 genes without replacement from the selected 3529 genes and generated three cell-types (1200 cells in total). We randomly generated gene counts for 400 cells in each cell-type. In order to generate cells with different DEG numbers, we randomly selected *n* genes (*n* = 50, 100 and 150) from the chosen 1000 genes without replacement and set the mean expression of these genes 1.5 times higher in one cluster than in the other two (1.5 is the median of the gene expression ratio between cells with “CA1 Pyramidal” and “Oligodendrocytes” labels in the dataset (Fig. S4)).

### Evaluation of clustering performance

#### Evaluation of clustering performance using simulated data sets with three genes and three clusters

We assumed the number of cell-types *K* = 3 was known in all algorithms. We performed K-means clustering and spectral clustering on imputed counts from published algorithms with the following transformations: log(1 + count), PCA on log(1 + count) with 2 components, PCA on log(1 + count) with components explaining 25% or 40% of variance. Since we do not know the transformation required to achieve best performance for published imputation algorithms, we tested all 8 transformations for each, and chose the one with the best score for comparison. We also ran the JOINT algorithm (initialized with the same 8 conditions) using original unimputed counts, and chose the one with the highest likelihood as the final solution. In order to obtain clustering scores for JOINT, we assigned each individual cell to the cell-type with the highest posterior probability, converting soft-clustering into hard-clustering results. Although Seurat process [15] can also be used for clustering, different parameters must be chosen for each individual dataset in order to achieve cluster number *K* = 3. Given that the performance of multiple algorithms at different dropout probabilities and DEG numbers needed to be tested extensively, K-means clustering method was used to simplify the process. It is also worth emphasizing that for data mapping and visualization in lower dimensional space, we applied the PCA from the original data without dropout, to the imputed data from published algorithms and data from JOINT, so that all data were transformed with the same projection from higher dimensional space to 2-dimensional space (Fig. 3, Fig. S6, and S7). Mapping to 2-dimensional space allows us to compare these different algorithms by inferring aspects of their relative positions in the original higher dimensional space. This is different than published work where PCA is performed for each individual dataset [11], which makes data incomparable following transformation. Although the simulated dataset may not have the same distribution as the original data, the performance of different algorithms in various conditions can be investigated.

#### Evaluation of clustering performance using real, large-scale scRNA-Seq datasets

We first applied Saver and scImpute algorithms to Baron and Zeisel datasets with default parameters for imputation. Then, we applied standard Seurat process with default parameters to the imputed data using 2000 highly expressed genes and cluster number *K* = 9 and 9 for each dataset. The number of PCA components in Seurat [15] was set to 25 and 45 (from the elbow method [15, 27]) for Baron and Zeisel datasets respectively. Finally, we applied the JOINT algorithm to both datasets.

#### Correlation analysis (cell and gene correlation)

We consider cell to cell correlation and gene to gene correlation. For cell to cell correlation, let *x*_*c*_ = [*x*_*c*,1_,..., *x*_*c*,*G*_]^*T*^ be a vector of counts without dropout for cell *c* and *y*_*c*_ = [*y*_*c*,1_,..., *y*_*c*,*G*_]^*T*^ be the corresponding vector of imputed counts. We compute the Pearson correlation between *x*_*c*_ and *y*_*c*_ as

$$ {\rho}_c= pearsonr\left({x}_c,{y}_c\right). $$

The cell to cell correlation is defined as the average of *ρ*_*c*_ across all cells, i.e.,

$$ {\rho}_{cell}=\frac{1}{C}\sum \limits_{c=1}^C{\rho}_c. $$

Similarly, *x*_*g*_ = [*x*_1,*g*_,..., *x*_*C*,*g*_]^*T*^ be a vector of counts without dropout for gene *g* and *y*_*c*_ = [*y*_1,*g*_,..., *y*_*C*,*g*_]^*T*^ be the corresponding vector of imputed counts. We compute the Pearson correlation between *x*_*g*_ and *y*_*g*_ as

$$ {\rho}_g= pearsonr\left({x}_g,{y}_g\right). $$

The gene to gene correlation is defined as the average of *ρ*_*g*_ across all gene

$$ {\rho}_{gene}=\frac{1}{G}\sum \limits_{g=1}^G{\rho}_g. $$

### Imputation algorithm for data visualization

We impute the observed counts directly. If the observed count is non-zero, we treat it as it is and do not perform imputation. If the observed count is zero, we impute it based on the posterior mean calculated from the JOINT algorithm. Consider a simple case in which we only have one cluster *K* = 1, one negative binomial component *L* = 2, and the observed count is 0. If the observed count is purely from the negative binomial component, the observed count 0 is the true count (the true expression is 0). If the observed count 0 is purely from the zero component, the best estimate in this case is the mean from negative binomial component which we assume is 5. If the probability that the 0 count is from the zero component *q*_*0*_ = 0.2, the probability from the negative binomial component 1-*q*_*0*_ = 0.8, and the mean of negative binomial component is 5, then the mean of the count imputed for given observed 0 is 0.2∗5 + 0.8∗0 = 1. We apply the idea formally, given observed count *x*_*c*_ in cell *c*, we first compute the posterior probability that *c* is from type *k* as

$$ p\left(k|{x}_c\right)=\frac{\pi_k{\Pi}_g{\sum}_l{q}_{g,k,l}h\left({x}_{c,g}|{\theta}_{g,k,l}\right)}{\sum \limits_{\kappa =0}^{K-1}{\pi}_{\kappa }{\Pi}_g{\sum}_{l^{\prime }}{q}_{g,\kappa, {l}^{\prime }}h\left({x}_{c,g}|{\theta}_{g,\kappa, {l}^{\prime }}\right)}, $$

where

$$ h\left({x}_{c,g}|{\alpha}_{g,k,l},{\beta}_{g,k,l}\right)=\left\{\begin{array}{ll}\int Gamma\left({\lambda}_{g,k,l}|{\alpha}_{g,k,l},{\beta}_{g,k,l}\right) Poisson\left({x}_{c,g}|{s}_c{\lambda}_{g,k,l}\right)d{\lambda}_{g,k,l}& l>0\\ {}1,& l=0\end{array}\right.. $$

Given *x*_*g*,*c*_ for gene *g* and cell-type *k*, the probability of *x*_*g*,*c*_ from the *l*-th negative binomial component is

$$ p\left(l|k,{x}_{g,c}\right)=\frac{q_{g,k,l}h\left({x}_{c,g}|{\theta}_{g,k,l}\right)}{\sum_{l^{\prime }}{q}_{g,k,{l}^{\prime }}h\left({x}_{c,g}|{\theta}_{g,k,{l}^{\prime }}\right)}. $$

The mean of each component *l* is *s*_*c*_*m*_*g*,*k*,*l*_ where

$$ {m}_{g,k,l}=\left\{\begin{array}{ll}\frac{\alpha_{g,k,l}}{\beta_{g,k,l}}& l>0\\ {}0,& l=0\end{array}\right. $$

With probability 1 − *p*(0|*k*, *x*_*g*,*c*_) the observed 0 is from a negative binomial component and we do not need imputation in this case. With probability *p*(0|*k*, *x*_*g*,*c*_) the observed count is from dropout events and we use the mean expression (conditional on this count is truly expressed) as the best estimate for imputation. The probability of *l* > 0 conditional on this count is truly expressed is

$$ p\left(l|k,{x}_{g,c}, expressed\right)=\frac{p\left(l|k,{x}_{g,c}\right)p\left( expressed|k,{x}_{g,c},l\right)}{p\left( expressed|k,{x}_{g,c}\right)}=\frac{p\left(l|k,{x}_{g,c}\right)}{p\left( expressed|k,{x}_{g,c}\right)}=\frac{p\left(l|k,{x}_{g,c}\right)}{1-p\left(0|k,{x}_{g,c}\right)}. $$

We thus have the imputation value as

$$ \sum \limits_kp\left(k|{x}_c\right)\left(1-p\left(0|k,{x}_{g,c}\right)\right)\ast 0+p\left(0|k,{x}_{g,c}\right)\sum \limits_{l>0}\frac{p\left(l|k,{x}_{g,c}\right)}{1-p\left(0|k,{x}_{g,c}\right)}{s}_c{m}_{g,k,l}={s}_c\sum \limits_kp\left(k|{x}_c\right)\frac{p\left(0|k,{x}_{g,c}\right)}{1-p\left(0|k,{x}_{g,c}\right)}\sum \limits_{l>0}p\left(l|k,{x}_{g,c}\right){m}_{g,k,l}. $$

### DEG analysis

We apply the Wald test [28] for DEG analysis by directly estimating the mean and the variance of expression conditional on that gene is expressed (or no dropout) for cell-type *k*. Given *p*(*k*|*x*_*c*_) and *p*(*l* = 0|*k, x*_*c,g*_), let *w*_*c*,*k*_ = *p*(*k*|*x*_*c*_) and *v*_*c*,*g*,*k*_ = 1 − *p*(*l* = 0|*k*, *x*_*c*,*g*_), where *v*_*c*,*g*,*k*_ is the probability that the observed zero-count is from a negative binomial component. We find the mean by minimizing

$$ \sum \limits_{c,{x}_{c,g}>0}{w}_{c,k}{\left|{x}_{c,g}-{m}_{g,k}\right|}^2+\sum \limits_{c,{x}_{c,g}==0}{w}_{c,k}{v}_{c,g,k}{\left|{x}_{c,g}-{m}_{g,k}\right|}^2. $$

We obtain

$$ {m}_{g,k}=E\left({x}_{c,\mathrm{g}}|k, expressed\right)=\frac{\sum \limits_{c,{x}_{c,g}>0}{w}_{c,k}{x}_{c,g}}{\sum \limits_{c,{x}_{c,g}>0}{w}_{c,k}+{\sum}_{c,{x}_{c,g}==0}{w}_{c,k}{v}_{c,g,k}}, $$

which is a weighted average with weight the probability of the observed count that is expressed in cell-type *k*. Similarly, we compute *E*(*x*^*2*^_*c*,*g*_|*k*) and obtain the variance as

$$ {\sigma}^2\left({x}_{c,g}|k, expressed\right)=E\left({x}_{c,g}^2|k, expressed\right)-{E}^2\left({x}_{c,g}|k, expressed\right). $$

Wald test [28] is used with the estimated mean and variance. After model training, it requires simple arithmetic operations to compute the mean and variance for Wald test. The Wald test *p*-values are adjusted using the Benjamini and Hochberg method [29]. As hard-clustering is a special case of soft-clustering with *p*(*k*|*x*_*c*_)∈{0, 1}, all the proposed DEG algorithms can be readily applied to hard-clustering as well. We are aware that we can use Fisher information matrix to estimate the variance of MLE estimate. However, although a closed-form of Fisher information matrix can be derived, we find the matrix is not always positive semidefinite for real scRNA-Seq data. Therefore, the MLE estimate method cannot be used directly to identify the variance of the EM algorithm. We can also use the likelihood-ratio test. However, it requires training the JOINT multiple times, which is computational expensive.

## Supplementary Information

**Additional file 1: Fig. S1.** Convergence of the JOINT algorithm with iterations. Convergence of *q*_*g,k,l*_ (**a**), *α*_*g,k,l*_ (**b**), *β*_*g,k,l*_ (**c**), and *π*_*k*_ (**d**) for different genes and cell clusters to true values with iterations.

**Additional file 2: Fig. S2.** Convergence of the JOINT algorithm with number of samples. Convergence of *q*_*g,k,l*_ (**a**), *α*_*g,k,l*_ (**b**), *β*_*g,k,l*_ (**c**), *π*_*k*_ (**d**), $\left(m\left(\mathbf{e},\left|{m}_{g,k}\hbox{-} {\hat{m}}_{g,k}\right|/{m}_{g,k}\right)\right.$, the mean of absolute difference between the theoretical mean from zero-inflated negative binomial model and the mean from model using estimated parameters over the theoretical mean), $\left({p}_0\left(\mathbf{f},\left|{p}_{g,k}^0\hbox{-} {\hat{p}}_{g,k}^0\right|/{p}_{g,k}^0\right)\right.$, the mean of absolute difference between the theoretical zero-count probability from zeroinflated negative binomial model and the zero-count probability from model using estimated parameters over the theoretical probability), $\left(\operatorname{var}\left(\mathbf{g},\left|{\mathit{\operatorname{var}}}_{g,k}\hbox{-} \mathrm{v}\hat{\mathrm{a}}{\mathrm{r}}_{g,k}\right|/{\mathit{\operatorname{var}}}_{g,k}\right)\right.$, the mean of absolute difference between the theoretical variance from zero-inflated negative binomial model and variance from model using estimated parameters over the theoretical variance) to true values with the number of samples. Error bars in (**a**) - (**d**) indicate the full range of data variation.

**Additional file 3: Fig. S3.** Convergence of the JOINT algorithm with dropout probabilities. Convergence of *q*_*g,k,l*_ (**a**), *α*_*g,k,l*_ (**b**), *β*_*g,k,l*_ (**c**), *π*_*k*_ (**d**), and $\left(m\left(\mathbf{e},\left|{m}_{g,k}\hbox{-} {\hat{m}}_{g,k}\right|/{m}_{g,k}\right)\right.$, i.e. the mean of absolute difference between the theoretical mean from zero-inflated negative binomial model and the mean from model using estimated parameters over the theoretical mean) to true values with dropout probabilities. Error bars in (**a**) - (**d**) indicate the full range of data variation.

**Additional file 4: Fig. S4.** The ratio of mean gene expression between pyramidal CA1 neurons and oligodendrocytes in the Zeisel dataset. (**a**) - (**b**) Histogram of *α* (**a**) and *β* (**b**) values for each gene when pyramidal CA1 neuron expression counts were used in model training. (**c**) Histogram of the ratio of mean gene expression between pyramidal CA1 neurons and oligodendrocytes. Note the median of the gene expression ratio between cells with “CA1 Pyramidal” and “Oligodendrocytes” labels in the Zeisel dataset is 1.5.

**Additional file 5: Fig. S5.** Simulated data at different dropout probabilities and DEG numbers. (**a**) Simulated datasets with three clusters when there is no dropout and DEG number set to 150, 100, and 50. (**b**) Simulated dataset with three clusters when dropout probability is set to 0.1, and DEG number set to 150, 100, and 50. (**c**) Simulated dataset with three clusters when dropout probability is set to 0.2, and DEG number set to 150, 100, and 50. (**d**) Simulated dataset with three clusters when dropout probability is set to 0.3, and DEG number set to 150, 100, and 50. (**e**) Simulated dataset with three clusters when dropout probability is set to 0.4, and DEG number set to 150, 100, and 50. For datasets with dropout, we applied the PCA from the original dataset without dropout to obtain the 2-dimensional plot. These simulated data show the impact of dropout probability and DEG number on the destruction of single-cell data.

**Additional file 6: Fig. S6.** Comparison of clustering performance of different algorithms at various dropout probabilities and DEG numbers. (**a**) Cell clustering by Saver, scImpute, and JOINT on a simulated dataset with three clusters (dropout probability set to 0.1 and DEG number set to 50). Original data without dropout is shown on the left. K-means clustering method is used for published imputation algorithms. Adjusted Rand Index for each algorithm is shown. Imputation algorithm in JOINT is used for data visualization. (**b**) Cell clustering by Saver, scImpute, and JOINT on a simulated dataset with three clusters (dropout probability set to 0.1 and DEG number set to 100). (**c**) Cell clustering by Saver, scImpute, and JOINT on a simulated dataset with three clusters (dropout probability set to 0.1 and DEG number set to 150). (**d**) Cell clustering scores are compared for Saver, scImpute, and JOINT algorithms at different dropout probabilities on a dataset with 100 DEG. (**e**) Correlation of cell clustering results from Saver, scImpute, and JOINT to original “true labels” averaged across all genes (Gene Correlation) or cells (Cell Correlation) at different dropout probabilities. Correlation coefficients generated from a dataset with 100 DEG are shown. (**f**) - (**g**) The JOINT algorithm determines cell cluster numbers automatically by likelihood (**f**) and AIC (**g**) tests. For each dataset, we applied the PCA from the original dataset without dropout to obtain the 2-dimensional plot.

**Additional file 7: Fig. S7.** Cell clustering data visualization by the JOINT imputation algorithm at different dropout probabilities and DEG numbers. (**a**) - (**d**) Cell clustering by JOINT on a simulated dataset with three clusters when dropout probability is set to 0.1 (**a**), 0.2 (**b**), 0.3 (**c**), and 0.4 (**d**), and DEG number set to 150, 100, and 50. For each dataset, we applied the PCA from the original dataset without dropout to obtain the 2- dimensional plot.

**Additional file 8: Fig. S8.** EM algorithm in JOINT improves the performance of cell clustering. (**a**) Clustering scores that JOINT obtained on the Zeisel dataset when the initial points were selected by the K-means method, with and without application of the EM algorithm. (**b**) Clustering scores that JOINT obtained on the Zeisel dataset when the initial points were randomly selected, with and without application of the EM algorithm.

**Additional file 9: Table S1.** Comparison of clustering performance for JOINT and published imputation algorithms on a simulated dataset.

**Additional file 10: Table S2.** Comparison of computing time when JOINT is run on GPU vs. CPU.

## Data Availability

Saver 1.1.2 was used in this study. Saver software can be found at https://github.com/mohuangx/SAVER. ScImpute 0.0.9 was used in this study. ScImpute software can be found at https://github.com/Vivianstats/scImpute. Seurat 3.1.4 was used in this study. Seurat software can be found at https://satijalab.org/seurat/. Three published scRNA-Seq datasets are used in this study: Baron (GSM2230757), Zeisel (http://linnarssonlab.org/cortex/), and Chu (GSE75748). JOINT code can be found at https://github.com/wanglab-georgetown/JOINT.
