# Correction to: JOINT for large-scale single-cell RNAsequencing analysis via soft-clustering and parallel computing

**DOI:** 10.1186/s12864-021-07408-5

**Published:** 2021-03-29

**Authors:** Tao Cui, Tingting Wang

**Affiliations:** 1 Department of Pharmacology and Physiology, Georgetown University Medical Center, Washington, DC 20057 USA; 2 Interdisciplinary Program in Neuroscience, Georgetown University Medical Center, Washington, DC 20057 USA

**Correction to: BMC Genomics 22, 47 (2021)**

**https://doi.org/10.1186/s12864-020-07302-6**

Following publication of the original article [1], several errors were identified in the “JOINT algorithm” and the “Imputation algorithm for data visualization” sub-sections, as well as in the Additional files and their captions.

In the “JOINT algorithm” sub-section the first two equations contained errors, which are shown below:

**Incorrect**

In the JOINT algorithm we consider a general mixture model

$$ {q}_{g,k,0}+\sum \limits_{l=1}^{L-1}{q}_{g,k,l}{\left(\frac{\beta_{g,k,l}}{\beta_{g,k,l}+{s}_c}\right)}^{\alpha_{g,k,l}} $$

where *x* is observed count number, *k* is the number of cell-types, *π*_*k*_ is the probability of choosing cell-type *k* and *f*_*k*_(*x*|*θ*_*k*_) is the probability of observing *x* given parameters *θ*_*k*_ in cell-type *k*. Given *x* and *θ*_*k*_, we compute the posterior probability of observed counts *x* from cell-type *k* as

$$ {\boldsymbol{m}}_{\boldsymbol{g},\boldsymbol{k},\boldsymbol{l}}=\left\{\begin{array}{cc}\frac{{\boldsymbol{\alpha}}_{\boldsymbol{g},\boldsymbol{k},\boldsymbol{l}}}{{\boldsymbol{\beta}}_{\boldsymbol{g},\boldsymbol{k},\boldsymbol{l}}}& \boldsymbol{l}>\mathbf{0}\\ {}\mathbf{0},& \boldsymbol{l}=\mathbf{0}\end{array}\right. $$

**Correct**

In the JOINT algorithm we consider a general mixture model

$$ \boldsymbol{p}\left(\boldsymbol{x}\right)=\sum \limits_{\boldsymbol{k}=\mathbf{0}}^{\boldsymbol{K}-\mathbf{1}}{\boldsymbol{\pi}}_{\boldsymbol{k}}{\boldsymbol{f}}_{\boldsymbol{k}\left(\boldsymbol{x}|{\boldsymbol{\theta}}_{\boldsymbol{k}}\right),} $$

where *x* is observed count number, *k* is the number of cell-types, *π*_*k*_ is the probability of choosing cell-type *k* and *f*_*k*_(*x*|*θ*_*k*_) is the probability of observing *x* given parameters *θ*_*k*_ in cell-type *k*. Given *x* and *θ*_*k*_, we compute the posterior probability of observed counts *x* from cell-type *k* as

$$ \boldsymbol{p}\left(\boldsymbol{k}|\boldsymbol{x}\right)=\frac{{\boldsymbol{\pi}}_{\boldsymbol{k}}{\boldsymbol{f}}_{\boldsymbol{k}}\left(\boldsymbol{x}|{\boldsymbol{\theta}}_{\boldsymbol{k}}\right)}{\sum_{\boldsymbol{k}=\boldsymbol{o}}^{\boldsymbol{K}-\mathbf{1}}{\boldsymbol{\pi}}_{\boldsymbol{k}}{\boldsymbol{f}}_{\boldsymbol{k}}\left(\boldsymbol{x}|{\boldsymbol{\theta}}_{\boldsymbol{k}}\right)}. $$

In the “Imputation algorithm for data visualization” sub-section the below equation was missing following the sentence “The mean of each component *l* is *s*_*c*_*m*_*g*,*k*,*l*_ where”:

$$ {\boldsymbol{m}}_{\boldsymbol{g},\boldsymbol{k},\boldsymbol{l}}=\left\{\begin{array}{cc}\frac{{\boldsymbol{\alpha}}_{\boldsymbol{g},\boldsymbol{k},\boldsymbol{l}}}{{\boldsymbol{\beta}}_{\boldsymbol{g},\boldsymbol{k},\boldsymbol{l}}}& \boldsymbol{l}>\mathbf{0}\\ {}\mathbf{0},& \boldsymbol{l}=\mathbf{0}\end{array}\right. $$

Finally, it was noted that there were several typographical errors in the Additional file captions in the PDF version of the published articles, which have been corrected.

The original article has been updated.
