# Supplementary figures and images for: JOINT for large-scale single-cell RNA-sequencing analysis via soft-clustering and parallel computing
Source: BMC Genomics. 2021 Jan 11;22:47. doi: 10.1186/s12864-020-07302-6 (PMC7798298; doi:10.1186/s12864-020-07302-6)

Fig. S1

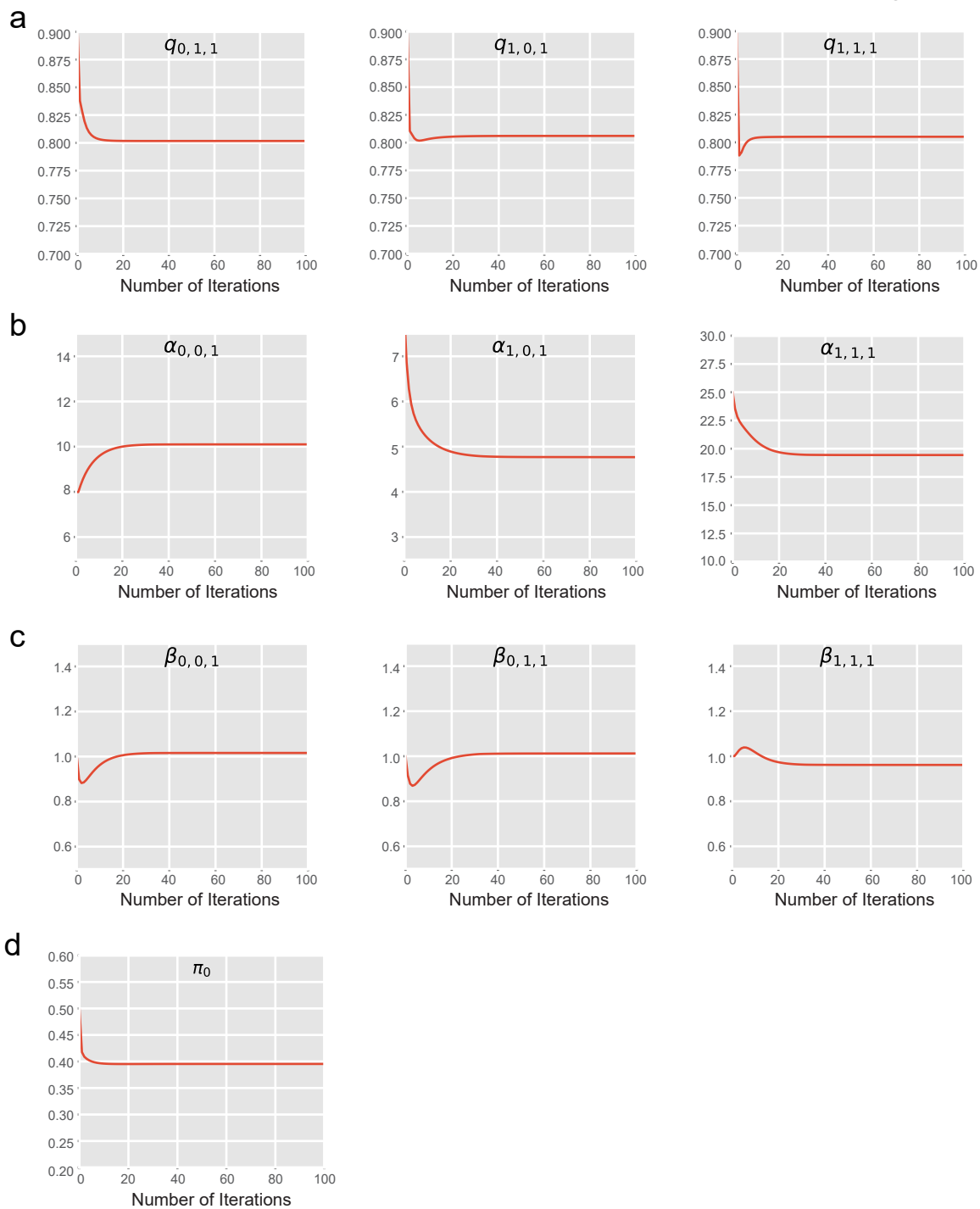

Supplement: Supplementary file 1 — Additional file 1: Fig. S1. Convergence of the JOINT algorithm with iterations. Convergence of qg,k,l (a), αg,k,l (b), βg,k,l (c), and πk (d) for different genes and cell clusters to true values with iterations. [file 12864_2020_7302_MOESM1_ESM.pdf]

Fig. S2

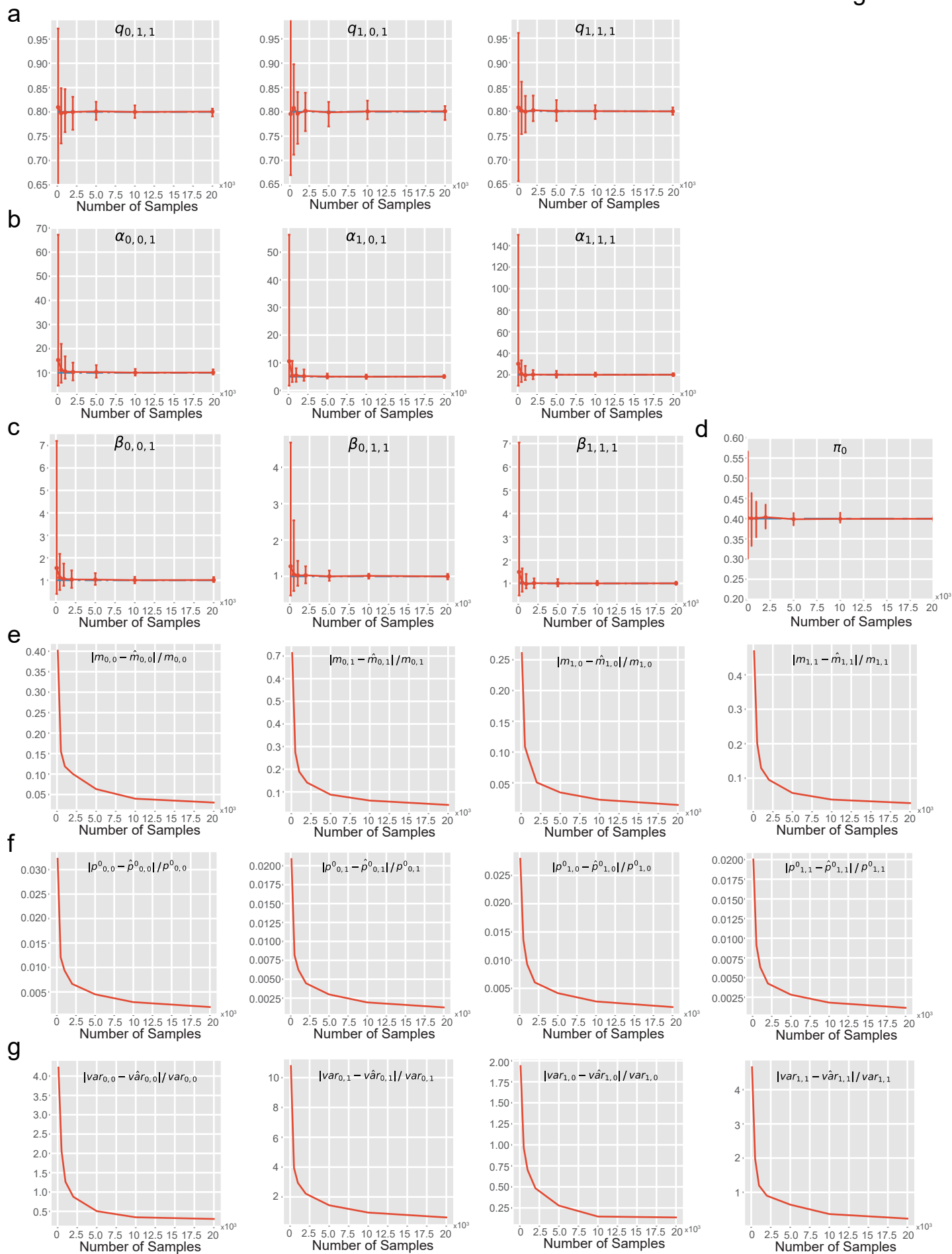

Supplement: Supplementary file 2 — Additional file 2: Fig. S2. Convergence of the JOINT algorithm with number of samples. Convergence of qg,k,l (a), αg,k,l (b), βg,k,l (c), πk (d), $\left(m\left(\mathbf{e},\left|{m}_{g,k}\hbox{-} {\hat{m}}_{g,k}\right|/{m}_{g,k}\right)\right.$memg,k‐m^g,k/mg,k, the mean of absolute difference between the theoretical mean from zero-inflated negative binomial model and the mean from model using estimated parameters over the theoretical mean), $\left({p}_0\left(\mathbf{f},\left|{p}_{g,k}^0\hbox{-} {\hat{p}}_{g,k}^0\right|/{p}_{g,k}^0\right)\right.$p0fpg,k0‐p^g,k0/pg,k0, the mean of absolute difference between the theoretical zero-count probability from zeroinflated negative binomial model and the zero-count probability from model using estimated parameters over the theoretical probability), $\left(\operatorname{var}\left(\mathbf{g},\left|{\mathit{\operatorname{var}}}_{g,k}\hbox{-} \mathrm{v}\hat{\mathrm{a}}{\mathrm{r}}_{g,k}\right|/{\mathit{\operatorname{var}}}_{g,k}\right)\right.$vargvarg,k‐va^rg,k/varg,k, the mean of absolute difference between the theoretical variance from zero-inflated negative binomial model and variance from model using estimated parameters over the theoretical variance) to true values with the number of samples. Error bars [file 12864_2020_7302_MOESM2_ESM.pdf]

Fig. S3

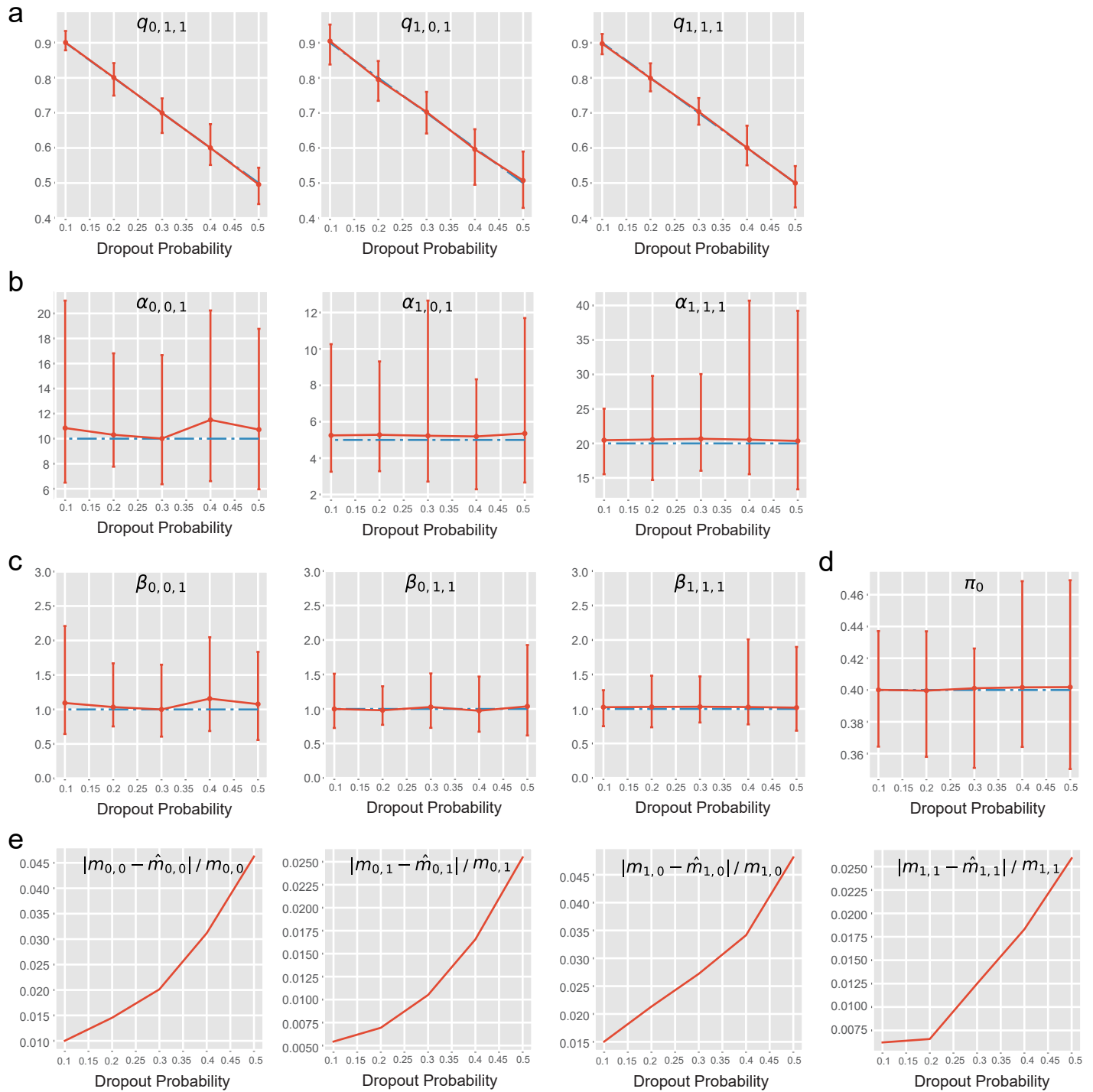

Supplement: Supplementary file 3 — Additional file 3: Fig. S3. Convergence of the JOINT algorithm with dropout probabilities. Convergence of qg,k,l (a), αg,k,l (b), βg,k,l (c), πk (d), and $\left(m\left(\mathbf{e},\left|{m}_{g,k}\hbox{-} {\hat{m}}_{g,k}\right|/{m}_{g,k}\right)\right.$memg,k‐m^g,k/mg,k, i.e. the mean of absolute difference between the theoretical mean from zero-inflated negative binomial model and the mean from model using estimated parameters over the theoretical mean) to true values with dropout probabilities. Error bars in (a) - (d) indicate the full range of data variation. [file 12864_2020_7302_MOESM3_ESM.pdf]

Fig. S4

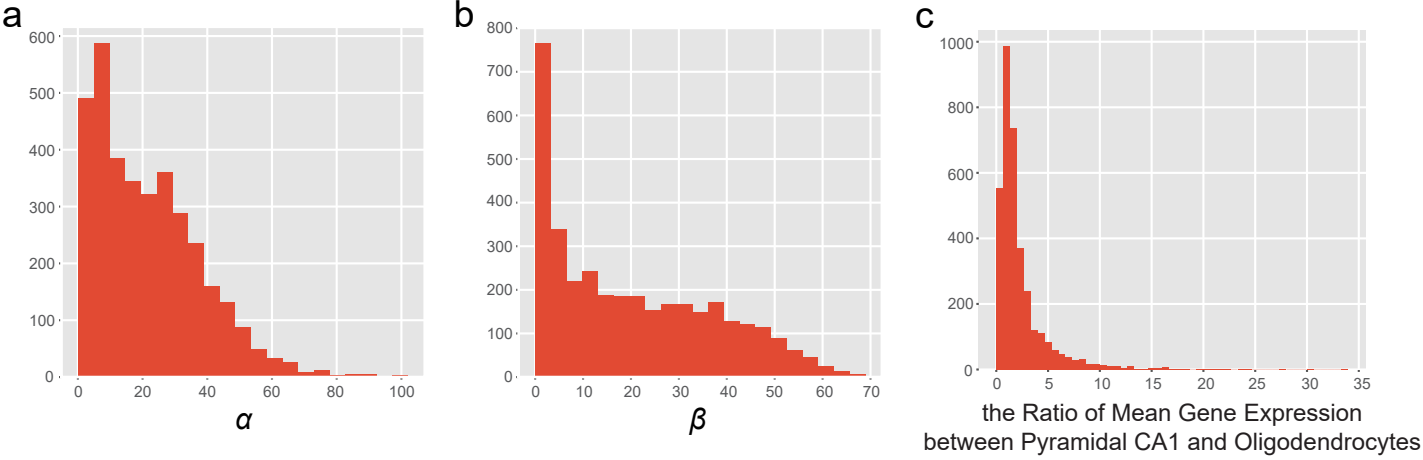

Supplement: Supplementary file 4 — Additional file 4: Fig. S4. The ratio of mean gene expression between pyramidal CA1 neurons and oligodendrocytes in the Zeisel dataset. (a) - (b) Histogram of α (a) and β (b) values for each gene when pyramidal CA1 neuron expression counts were used in model training. (c) Histogram of the ratio of mean gene expression between pyramidal CA1 neurons and oligodendrocytes. Note the median of the gene expression ratio between cells with “CA1 Pyramidal” and “Oligodendrocytes” labels in the Zeisel dataset is 1.5. [file 12864_2020_7302_MOESM4_ESM.pdf]

Fig. S5

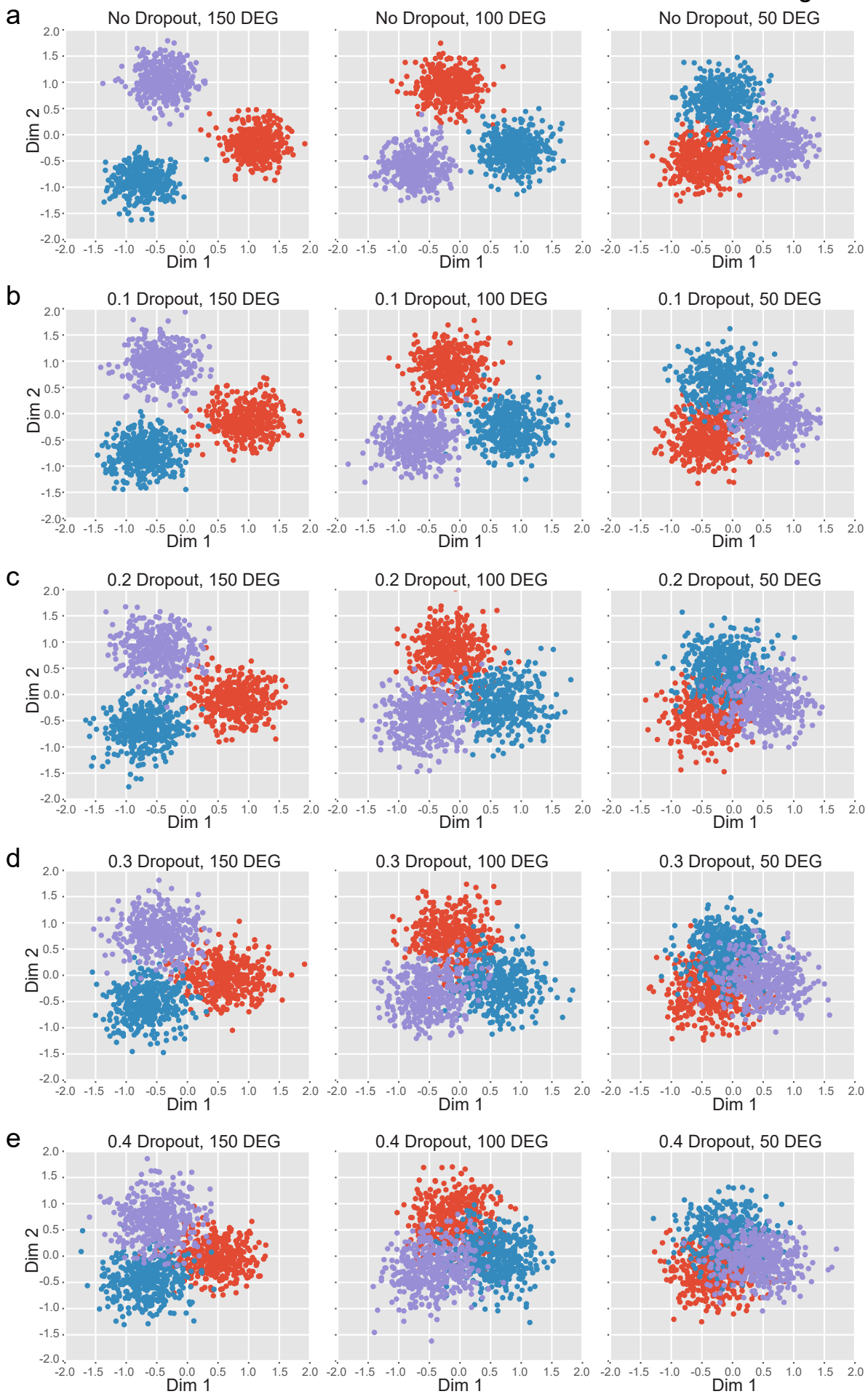

Supplement: Supplementary file 5 — Additional file 5: Fig. S5. Simulated data at different dropout probabilities and DEG numbers. (a) Simulated datasets with three clusters when there is no dropout and DEG number set to 150, 100, and 50. (b) Simulated dataset with three clusters when dropout probability is set to 0.1, and DEG number set to 150, 100, and 50. (c) Simulated dataset with three clusters when dropout probability is set to 0.2, and DEG number set to 150, 100, and 50. (d) Simulated dataset with three clusters when dropout probability is set to 0.3, and DEG number set to 150, 100, and 50. (e) Simulated dataset with three clusters when dropout probability is set to 0.4, and DEG number set to 150, 100, and 50. For datasets with dropout, we applied the PCA from the original dataset without dropout to obtain the 2-dimensional plot. These simulated data show the impact of dropout probability and DEG number on the destruction of single-cell data. [file 12864_2020_7302_MOESM5_ESM.pdf]

Fig. S6

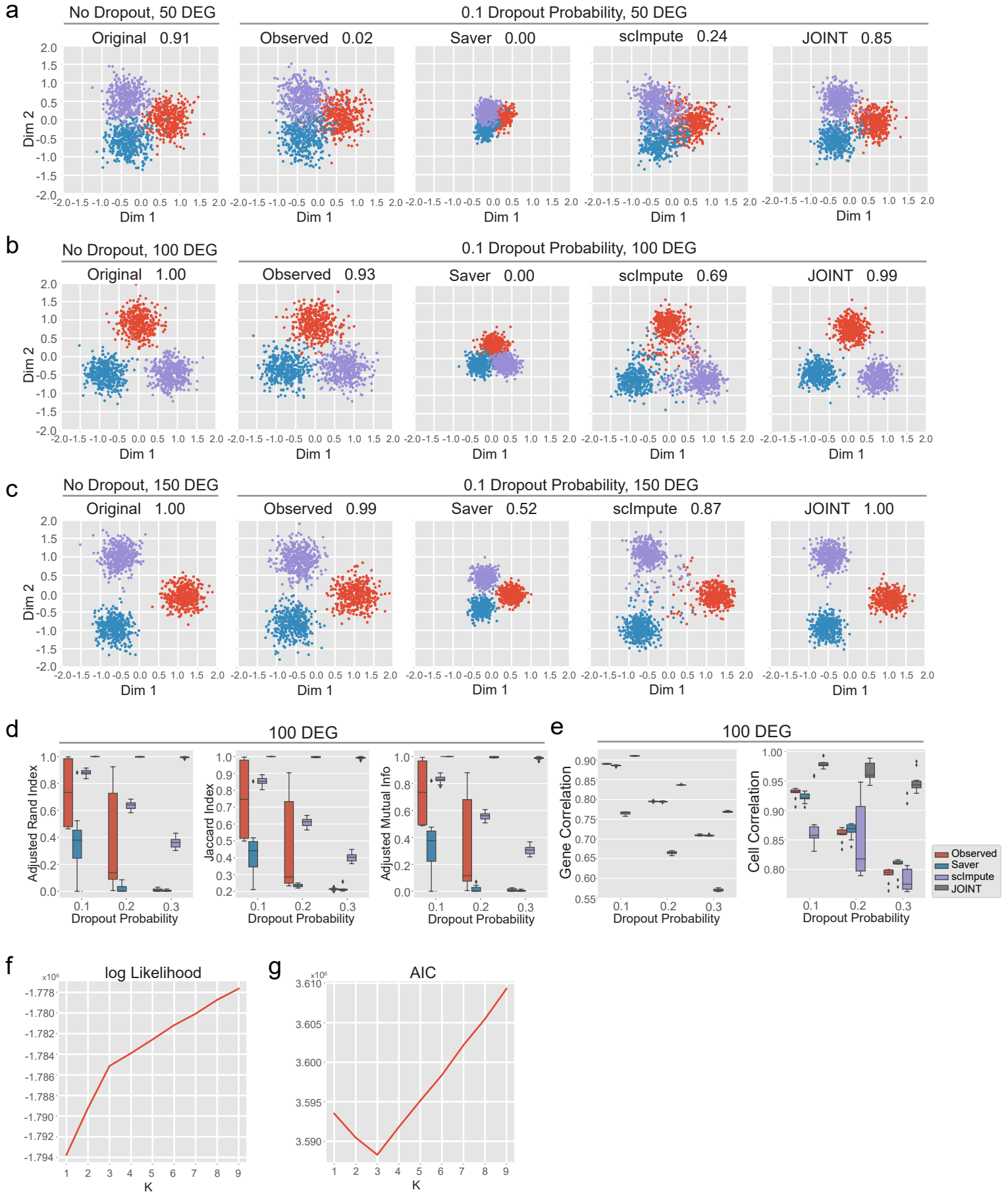

Supplement: Supplementary file 6 — Additional file 6: Fig. S6. Comparison of clustering performance of different algorithms at various dropout probabilities and DEG numbers. (a) Cell clustering by Saver, scImpute, and JOINT on a simulated dataset with three clusters (dropout probability set to 0.1 and DEG number set to 50). Original data without dropout is shown on the left. K-means clustering method is used for published imputation algorithms. Adjusted Rand Index for each algorithm is shown. Imputation algorithm in JOINT is used for data visualization. (b) Cell clustering by Saver, scImpute, and JOINT on a simulated dataset with three clusters (dropout probability set to 0.1 and DEG number set to 100). (c) Cell clustering by Saver, scImpute, and JOINT on a simulated dataset with three clusters (dropout probability set to 0.1 and DEG number set to 150). (d) Cell clustering scores are compared for Saver, scImpute, and JOINT algorithms at different dropout probabilities on a dataset with 100 DEG. (e) Correlation of cell clustering results from Saver, scImpute, and JOINT to original “true labels” averaged across all genes (Gene Correlation) or cells (Cell Correlation) at different dropout probabilities. Correlation coefficients generated from a dataset with 100 DEG are shown. (f) - (g) The JOINT algorithm determines cell cluster numbers automatically by likelihood (f) and AIC (g) tests. For each dataset, we applied the PCA from the original dataset without dropout to obtain the 2-dimensional plot. [file 12864_2020_7302_MOESM6_ESM.pdf]

Fig. S7

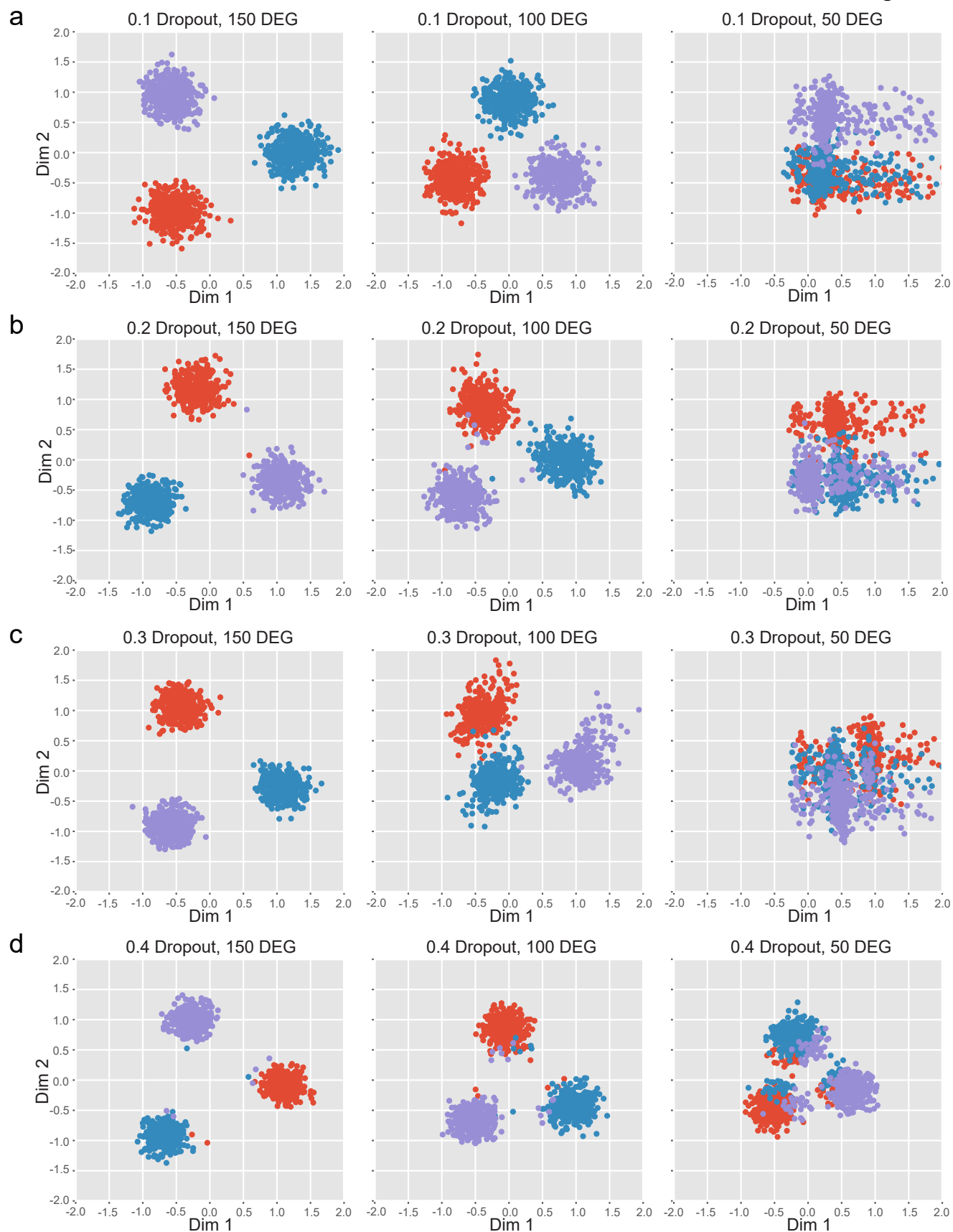

Supplement: Supplementary file 7 — Additional file 7: Fig. S7. Cell clustering data visualization by the JOINT imputation algorithm at different dropout probabilities and DEG numbers. (a) - (d) Cell clustering by JOINT on a simulated dataset with three clusters when dropout probability is set to 0.1 (a), 0.2 (b), 0.3 (c), and 0.4 (d), and DEG number set to 150, 100, and 50. For each dataset, we applied the PCA from the original dataset without dropout to obtain the 2- dimensional plot. [file 12864_2020_7302_MOESM7_ESM.pdf]

Fig. S8

**a**

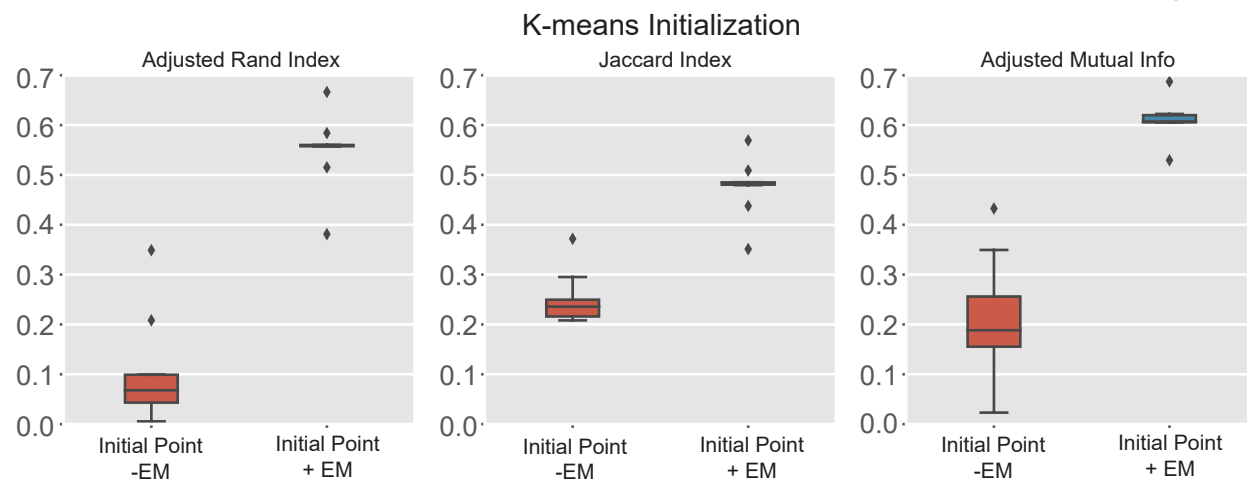

**b**

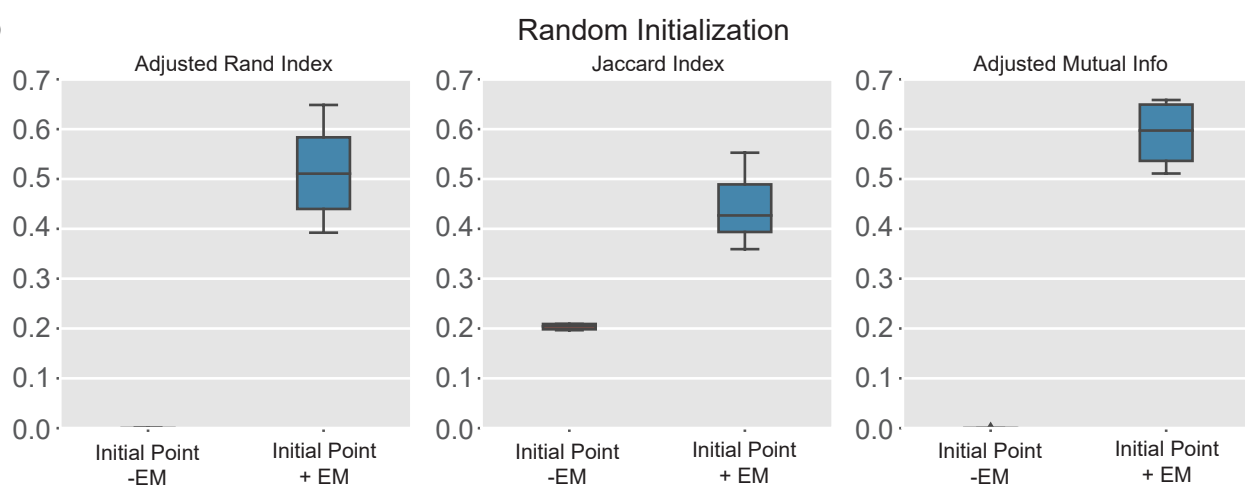

Supplement: Supplementary file 8 — Additional file 8: Fig. S8. EM algorithm in JOINT improves the performance of cell clustering. (a) Clustering scores that JOINT obtained on the Zeisel dataset when the initial points were selected by the K-means method, with and without application of the EM algorithm. (b) Clustering scores that JOINT obtained on the Zeisel dataset when the initial points were randomly selected, with and without application of the EM algorithm. [file 12864_2020_7302_MOESM8_ESM.pdf]
